# Impact of co-transfer of embryos produced by somatic cell nuclear transfer using two types of donor cells on pregnancy outcomes in dogs

**DOI:** 10.5713/ab.22.0043

**Published:** 2022-05-02

**Authors:** Young-Bum Son, Yeon Ik Jeong, Yeon Woo Jeong, Mohammad Shamim Hossein, Woo Suk Hwang

**Affiliations:** 1 Abu Dhabi Research Foundation, Seoul 08359, Korea; 2 UAE Biotech Research Center, P.O. Box 30310, Al Wathba, Abu Dhabi, United Arab Emirates; 3 Department of Companion Animal and Animal Resources Science, Joongbu University, Geumsan-gun, 32713, Korea; 4 Department of Biology, North-Eastern Federal University, Yakutsk, 67707, Sakha Republic, Russia

**Keywords:** Co-transfer, Dog, Pregnancy Outcome, Single Transfer, Somatic Cell Nuclear Transfer

## Abstract

**Objective:**

The present study analyzed the influence of co-transferring embryos with high and low cloning efficiencies produced via somatic cell nuclear transfer (SCNT) on pregnancy outcomes in dogs.

**Methods:**

Cloned dogs were produced by SCNT using donor cells derived from a Tibetan Mastiff (TM) and Toy Poodle (TP). The *in vivo* developmental capacity of cloned embryos was evaluated. The pregnancy and parturition rates were determined following single transfer of 284 fused oocytes into 21 surrogates and co-transfer of 47 fused oocytes into four surrogates.

**Results:**

When cloned embryos produced using a single type of donor cell were transferred into surrogates, the pregnancy and live birth rates were significantly higher following transfer of embryos produced using TP donor cells than following transfer of embryos produced using TM donor cells. Next, pregnancy and live birth rates were compared following single and co-transfer of these cloned embryos. The pregnancy and live birth rates were similar upon co-transfer of embryos and single transfer of embryos produced using TP donor cells but were significantly lower upon single transfer of embryos produced using TM donor cells. Furthermore, the parturition rate for TM dogs and the percentage of these dogs that remained alive until weaning was significantly higher upon co-transfer than upon single transfer of embryos. However, there was no difference between the two embryo transfer methods for TP dogs. The mean birth weight of cloned TM dogs was significantly higher upon single transfer than upon co-transfer of embryos. However, the body weight of TM dogs did not significantly differ between the two embryo transfer methods after day 5.

**Conclusion:**

For cloned embryos with a lower developmental competence, the parturition rate and percentage of dogs that remain alive until weaning are increased when they are co-transferred with cloned embryos with a greater developmental competence.

## INTRODUCTION

After the first cloned dog was produced by somatic cell nuclear transfer (SCNT) using oocytes retrieved by flushing, several studies reported cloning of dogs [1,2]. For a long time, dogs have maintained a close relationship with humans as companion animals and shared living environments. Consequently, dogs of various breeds and ages have been cloned for commercial purposes. Furthermore, dogs have similar physiological characteristics as humans, and it has been reported that more than 370 genes shared between dogs and humans are closely related to the mechanisms of human diseases [2]. Accordingly, several studies have used dogs to model various human diseases including diabetes and Alzheimer’s disease [3,4].

Dogs have unique reproductive characteristics compared with other mammals. They are non-seasonal and monoestrus animals, and their estrus interval is longer than that of other animals [5]. It has also been reported that oocytes ovulate in the metaphase I stage and mature into the metaphase II stage in the fallopian tube, and that there is heterogeneity in oocyte maturation in dogs [6]. These characteristics hamper cloning of dogs and thus studies of dog cloning are limited.

*In vivo* matured oocytes are crucial for dog cloning. *In vitro* maturation (IVM) and *in vitro* culture (IVC) protocols are well-established for animals such as cattle and pigs because a sufficient number of oocytes can be obtained from slaughterhouses [1,7]. However, oocytes of dogs are not readily available, and IVM and IVC of these oocytes are poorly studied and lack efficiency [8]. Therefore, dogs have been cloned by surgically transferring fused embryos, rather than blastocysts, which is generally performed to clone other mammals [1–4]. Studies concerning superovulation of oocyte donors, synchronization of surrogates, and techniques for retrieval and transfer of oocytes have been conducted to enhance the efficiency of cloned dog production [9–13]. In this study, a technique was evaluated to enhance the pregnancy and parturition rates upon transfer of cloned embryos with a low developmental capacity.

Several studies reported that the development and preg nancy efficiency of cloned embryos differ using cells from different donors [14,15]. The cloning efficiency is influenced by donor cell characteristics including cell passage, the cell cycle, and donor cell type. Among those factors, variation in the genetic features of cells from different donors is a crucial factor that affects pregnancy outcomes [14]. However, no study has investigated pregnancy and live birth rates upon co-transfer of embryos with different cloning efficiencies generated using different donor cells. Upon co-transfer of poor- and good-quality embryos, the former do not interfere with implantation of the latter [16]. Additionally, it was reported that the pregnancy and live birth rates do not differ following co-transfer of good- and poor-quality embryos and transfer of good-quality embryos alone at the cleavage stage [17,18]. The present study evaluated the impact of co-transfer of embryos generated using different donor cells and with different cloning efficiencies into homogenous surrogates on pregnancy outcomes.

## MATERIALS AND METHODS

### Animals

All animal experiments were performed according to the animal study guidelines approved by the ethics committee of the Abu Dhabi Biotech Research Foundation, Korea (Permit no. C-2013-01). In total, 64 oocyte donors and 25 surrogates were used. Female mixed breed dogs aged 3 to 5 years (body weight, 20 to 25 kg) were housed in indoor kennels (2.5×1.5 m) on a 12 h/12 h light/dark cycle with natural light, fed standard commercial dog food once daily, and given water *ad libitum*.

### Chemicals

All chemicals were purchased from Sigma (St. Louis, MO, USA), unless otherwise stated.

### Collection of oocytes

The estrus of bitches was monitored weekly by observing vulvar bleeding to detect the onset of the heat period. A blood sample (2 mL) was collected every day at the same time by cephalic venipuncture, and serum progesterone levels were measured using an electrochemiluminescence immunoassay (Cobas e411; Roche Diagnostics, Mannheim, Germany) with intra- and inter-assay coefficients of variation <4%. Ovarian ultrasonography was performed twice daily when serum progesterone levels exceeded 2 ng/mL. Ovulation was further verified by the rupture of follicles detected by transabdominal ultrasonography [8].

### Establishment of donor cells

All donor cells were established using a previously described protocol with minor modifications [8]. They were established from Tibetan Mastiff (TM) and Toy Poodle (TP) dogs with different fur colors and body sizes to clearly confirm the origin of the puppies. In brief, samples were obtained from the inguinal portions of the skin of a 5-year-old female TM and TP under light sedation using Zoletil 50 (Virbac, Carros, France). Sections of the subcutaneous tissues were washed twice with phosphate-buffered saline (Invitrogen, Carlsbad, CA, USA) and minced with a surgical blade on a culture dish (Becton Dickinson, Franklin Lincoln, NJ, USA). The minced tissues were dissociated with 0.25% trypsin-ethylenediaminetetraacetic acid solution (Invitrogen, USA) for 3 min. Trypsinized cells were washed twice by centrifugation at 300×g for 5 min and seeded onto 100 mm plastic culture dishes. Cells were subsequently cultured in 60 mm plastic culture dishes in the presence of fetal bovine serum (FBS; Invitrogen, USA), 1 mM sodium pyruvate, 1% (v/v) non-essential amino acids (Invitrogen, USA), and 1% antibiotic-antimycotic solution (Thermo Fisher Scientific, Waltham, MA, USA) at 37°C in a humidified atmosphere of 5% CO_2_ and 95% air. When cells became confluent, they were collected by trypsinization and frozen in Dulbecco’s modified eagle’s medium supplemented with 20% FBS and 10% dimethyl sulfoxide.

### Somatic cell nuclear transfer

SCNT was performed as described previously with minor modifications [8]. In brief, metaphase II oocytes were stripped from cumulus cells and enucleated by squeezing out the first polar body and the metaphase II plate in a small amount of surrounding cytoplasm using a glass pipette. All oocytes were pre-stained with 5 mg/mL bisbenzimide (Hoechst 33342). A trypsinized fibroblast with a smooth surface was transferred into the perivitelline space of an enucleated oocyte using a fine glass pipette. The couplets were equilibrated with 0.26 M mannitol solution containing 0.5 mM 4-(2-hydroxyethyl)-1-piperazineethanesulfonic acid, 0.1 mM CaCl_2_, and MgSO_4_ for 4 min, and then fused with two direct current pulses of 1.75 to 1.85 kV/cm for 15 μs using a BTX Electro-Cell Manipulator 2001 (BTX, San Diego, CA, USA).

### Embryo transfer

After fusion and activation, reconstructed embryos were loaded into a Tomcat catheter (Sherwood Medical, St. Louis, MO, USA) with 2 to 4 μL transfer medium and gently transferred into the distal two-thirds position of the oviduct without insufflating air. Anesthesia was induced with a mixture of xylazine hydrochloride (Bayer Korea, Ansan, Korea; 1 mg/kg body weight) ketamine HCl (YuHan Corporation, Seoul, KR; 4 mg/kg body weight) and maintained with isoflurane inhalational. The ovary with the greater amount of corpus luteum was approached via ventral laparotomy. The fat layer covering the ovary was gently grasped with forceps and suspended with a suture to exteriorize the end of the oviduct with fimbriae. The same number of embryos was transferred upon single transfer and co-transfer. For co-transfer, the same number of cloned embryos produced using each type of donor cell was transferred.

### Pregnancy diagnosis and measurement of body weight

Pregnancy was diagnosed as described previously with minor modifications [19]. Briefly, one veterinarian confirmed the pregnancy using real-time ultrasonography at 30 days after embryo transfer. Two-dimensional, gray-scale, real-time ultrasound images were produced using mechanical and phased-array sectors from a curved-linear transducer with a frequency of 3.5 MHz (Samsung Medison, Seoul, Korea). When no fetal heartbeat was observed, the fetus was obtained via an elective cesarean section to determine which type of donor cell it was established from. In the last week of pregnancy, the surrogate’s rectal temperature was monitored once or twice daily, and vulva discharge and other signs of impending parturition (anorexia, nesting, and lactation) were examined. The body weights of dogs were recorded for 40 days after birth. To prevent physiological changes affecting the body weights of dogs, they were measured at the same time (10:00 am) each day before feeding.

### Statistical analysis

Data were analyzed using SPSS for Windows (version 15; SPSS Inc., Chicago, IL, USA). Graphs were generated using GraphPad Prism (version 4.0). The fusion rate and average number of transferred embryos were compared between the groups using Tukey’s multiple range test. The birth weights and weight changes of cloned dogs were assessed using the independent T-test. The pregnancy rate was compared between the groups using Pearson’s Chi-square test and Fisher’s exact test. Data are represented as mean±standard deviation. A p value less than 0.05 was considered significant.

## RESULTS

### Comparison of the fusion rate and perinatal and pre-weaning development of cloned embryos generated using different donor cells

**Figure 1:**
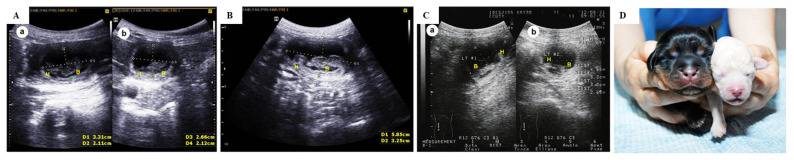
Representative ultrasonography images and a representative photograph of cloned dogs. Ultrasonography images of (A) twin fetuses following single transfer of embryos generated using TP donor cells, (B) a singleton fetus following single transfer of embryos generated using TM donor cells, and (C) twin fetuses following co-transfer of embryos generated using TP and TM donor cells on day 30. (D) A photograph of cloned puppies born following co-transfer of embryos generated using TP and TM donor cells. TP, Toy Poodle; TM, Tibetan Mastiff.

The present study compared co-transfer and single transfer of cloned embryos with different cloning efficiencies generated using different donor cells. Embryos were cloned using donor nuclei obtained from cells derived from skin of a TM and TP, which were called the TM and TP groups, respectively. Data about nuclear transfer, embryo transfer, and pregnancy outcomes upon single transfer of these cloned embryos are presented in Tables 1 and 2. The fusion rate was significantly higher in the TP group than in the TM group (Table 1). The pregnancy and live birth rates were significantly higher in the TP group than in the TM group (Table 2). Furthermore, the percentage of dogs that remained alive until weaning was significantly higher in the TP group than in the TM group (Table 2). The number of embryos transferred into surrogates did not significantly differ between the two groups (Table 2). A representative photograph of cloned dogs and representative ultrasonography images of TM and TP puppies are shown in Figure 1.

### Comparison of perinatal and pre-weaning development following single transfer and co-transfer of embryos

**Figure 2:**
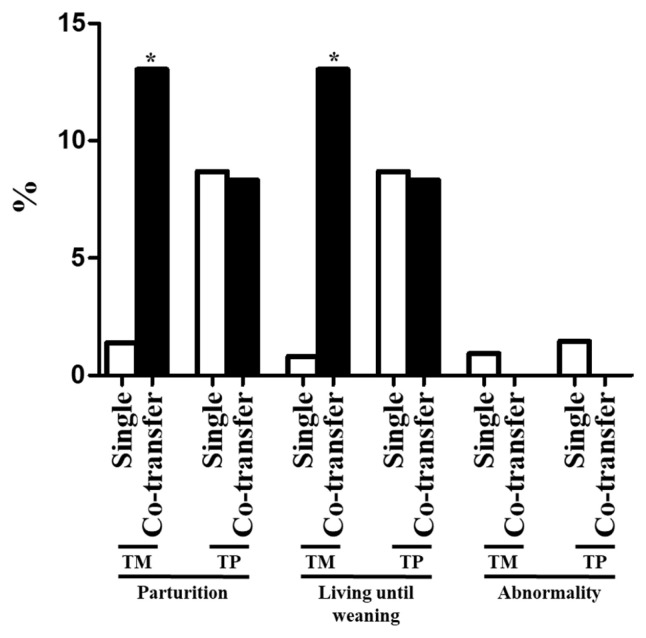
Parturition and abnormality rates and percentages of dogs that remained alive until weaning following single and co-transfer of cloned embryos generated using TM and TP donor cells. An asterisk represents a significant (p<0.05) difference. TM, Tibetan Mastiff; TP, Toy Poodle.

The pregnancy and live birth rates and percentage of dogs that remained alive until weaning are summarized in Table 2 and Figure 2. Each of these variables was significantly higher following co-transfer of embryos than following single transfer of embryos generated using TM donor cells (Table 2). Furthermore, the parturition rate and percentage of dogs that remained alive until weaning in the TM group, in which the cloning efficiency was low, were significantly increased by co-transfer of embryos (Figure 2). In the TP group, the parturition rate and percentage of dogs that remained alive until weaning did not significantly differ between the two embryo transfer methods (Figure 2). The percentage of dogs with abnormalities did not differ between the two embryo transfer methods; however, no abnormalities were observed after co-transfer of embryos (Table 2). The number of transferred embryos did not differ upon single transfer and co-transfer (Table 2).

### Comparison of birth and body weights of dogs following single transfer and co-transfer of embryos

**Figure 3:**
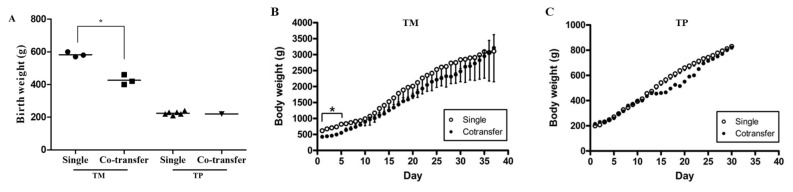
Birth weights and body weight changes over time of dogs born following single and co-transfer of embryos generated using TM and TP donor cells. (A) The birth weight of TM puppies was significantly lower (p<0.05) following co-transfer than following single transfer of embryos. (B and C) The body weight of cloned TM dogs was significantly (p<0.05) higher following single transfer than following co-transfer of embryos until the fifth day after birth. An asterisk (*) represents a significant (p<0.05) difference. TM, Tibetan Mastiff; TP, Toy Poodle.

We measured the birth weights of cloned dogs and their body weights over time (Figure 3). The body weights of TM dogs significantly differed between the two embryo transfer methods from day 0 to day 5, but not at later time points. The birth weights of TP dogs and their body weights over time did not significantly differ between the two embryo transfer methods.

## DISCUSSION

In the present study, we performed SCNT using two types of donor cells, transferred the resultant embryos into surrogates separately or simultaneously, and determined the pregnancy and parturition rates and percentage of dogs that remained alive until weaning. Embryos were transferred immediately after fusion and activation. Homogenous surrogates received similar numbers of cloned embryos upon single transfer and co-transfer. This study compared the outcomes following single transfer and co-transfer of embryos generated using two types of donor cells and with different cloning efficiencies for the first time.

It has been reported that the donor cell used as the oocyte nucleus in SCNT affects embryo development and pregnancy outcomes [14,20]. Several studies have investigated the development of SCNT embryos to improve the cloning efficiency [21,22]. However, no study has analyzed *in vitro* development of SCNT embryos dogs. Furthermore, it is technically difficult to recover embryos from fallopian tubes after transfer and artificial insemination at the pre-implantation stage [1]. Therefore, research about implantation and pregnancy after embryo transfer in dogs is limited. Owing to the unique reproductive characteristics of dogs, many studies have investigated the physiological characteristics of suitable surrogates, synchronization, and number and quality of embryos transferred into surrogates [12,23]. However, the low cloning efficiency achieved using cells derived from some donors hampers dog cloning. The present study sought to overcome this problem for large-scale cloning. The fusion rates and pregnancy and live birth rates following embryo transfer were determined. These rates significantly differed between cloned embryos produced using two types of donor cells.

Co-transfer of parthenogenotes and fertilized embryos with a high developmental capacity enhances the *in vivo* developmental capacity in recipients [24,25]. This demonstrates that there is a synergistic effect between embryos upon implantation. In pig and mouse, co-transfer of SCNT embryos and parthenogenetic embryos helps to initiate and maintain a pregnancy [24,25]. Several studies have evaluated the influence of embryo co-transfer into surrogates. Upon co-transfer of fertilized embryos with different cloning efficiencies, embryos with a low cloning efficiency exhibit higher implantation and pregnancy rates than upon single transfer, while embryos with a high cloning efficiency exhibit similar implantation and pregnancy rates as upon single transfer [16]. In humans, embryos produced from cryopreserved and fresh oocytes were co-transferred into recipients [26]. The implantation and pregnancy rates were higher upon co-transfer than upon single transfer of embryos produced from cryopreserved oocytes [26]. Our results are consistent with these previous reports. In the present study, the parturition rate and percentage of dogs that remained alive until weaning were significantly higher when embryos with a low cloning efficiency were co-transferred with embryos with a high cloning efficiency than when the former embryos were transferred alone (Figure 2). Meanwhile, the parturition rate and percentage of dogs that remained alive until weaning were similar upon co-transfer and single transfer of embryos with a high cloning efficiency (Figure 2). In SCNT, abnormal reprogramming of the donor nucleus by oocyte cytoplasm induces alterations of epigenetic modifications of key regulatory genes required for normal fetal and placental development [27]. Overall, our results showed that co-transfer of embryos with low and high cloning efficiencies might affect *in vivo* reprogramming of cloned embryos and improve parturition rates.

Abnormal reprogramming and DNA methylation follow ing SCNT can cause abortion, stillbirth, and death after birth [27]. Furthermore, cloned animals have a longer gestation period than non-cloned animal and exhibit abnormalities such as rapid fetal weight gain at the end of pregnancy, dyspnea, hyper-muscular dystrophy, and large size [19,28]. However, several studies reported that cloned animals have similar growth curves and health statuses as non-cloned animals [29,30]. Therefore, we evaluated birth weights, body weight changes over time, and abnormality rates of cloned dogs born after co-transfer and single transfer of embryos. Abnormalities such as abortion and hyper-muscular hypertrophy were observed following single transfer, but not co-transfer, of embryos. The birth weights of TM dogs were significantly lower after co-transfer than after single transfer of embryos, but their body weights did not differ between the two embryo transfer methods after 5 days. A previous study reported that birth weight is lower following a multiple pregnancy than following a single pregnancy [19]. Consistently, the body weight of TM dogs was lower after co-transfer than after single transfer of embryos from day 0 to day 5 (Figure 3). However, the body weights of these dogs did not significantly differ between the two embryo transfer methods after day 5 and their growth curves were normal (Figure 3).

In conclusion, the pregnancy and live birth rates and per centage of dogs that remained alive until weaning significantly differed following transfer of embryos generated using two types of donor cells. The parturition rate and percentage of dogs that remained alive until weaning were significantly higher when embryos generated using TM donor cells, which had a low cloning efficiency, and TP donor cells, which had a high cloning efficiency, were co-transferred into surrogates than when the former embryos were transferred alone. The cloned dogs had similar weight change curves until weaning. Although further studies of embryo reprogramming and DNA methylation during *in vivo* development are essential, we argue that pregnancy outcomes are better upon co-transfer of SCNT embryos with high and low cloning efficiencies than upon transfer of the latter embryos alone.

## Figures and Tables

**Table 1:** Effect of the type of donor cells used for SCNT on the fusion rate

Transfer method	Nuclear transfer				

	Donor cell	No. of dogs from which oocytes were retrieved	No. of oocytes		

			Retrieved	Reconstructed	Fused and transferred (%)^1)^
Single transfer	TM	45	520	460	215 (46.74±11.08)^a^
	TP	9	130	110	69 (62.73±8.46)^b^
Co-transfer	TM	5	48	36	17 (47.22±3.52)^a^
	TP	5	50	42	30 (71.43±5.27)^b^

SCNT, somatic cell nuclear transfer; TM, Tibetan Mastiff; TP, Toy Poodle.

1) The fusion rate was calculated as the percentage of reconstructed oocytes that underwent fusion.

a,b Different superscript letters within the same transfer method represent significance (p<0.05).

**Table 2:** Effect of the type of donor cells and transfer method on perinatal development and parturition

Transfer method	Embryo transfer						Parturition			
	
	Donor cell	No. of transferred embryos	No. of surrogates	Average number of transferred embryos per surrogate	No. of pregnancies (%)^1)^		No. of live births (%)^2)^			
	
					At mid-term	At term	At mid-term	At term	Abnormal	Alive until weaning
Single transfer	TM	215	16	13.43±1.59	5 (31.30)	3 (18.75)^a^	5 (2.33)^a^	3 (1.40)^a^	2 (0.93)	2 (0.93)^a^
	TP	69	5	13.80±0.84	4 (80.00)	4 (80.00)^b^	6 (8.70)^b^	6 (8.70)^b^	1 (1.45)	6 (8.70)^b^
Co-transfer	TM/TP	47	4	11.75±0.50	2 (50.00)	2 (50.00)^b^	4 (8.51)^ab^	4 (8.51)^b^	0 (0.00)	4 (8.51)^b^

TM, Tibetan Mastiff; TP, Toy Poodle.

1) The pregnancy rate was calculated as the percentage of surrogates that became pregnant.

2) The live birth rate was calculated as the percentage of transferred embryos that developed into puppies that were alive at birth.

a,b Different superscript letters represent significance (p<0.05).
